# Engineering Grain Architecture in Epitaxial Aluminum on Miscut Substrates Toward Various Clean Limits and Giant Superconductivity Modulation

**DOI:** 10.1002/smll.202512268

**Published:** 2026-01-14

**Authors:** Thi‐Hien Do, Pei‐Tzu Wu, Yu‐Yao Gao, Ching‐Hung Chen, Chu‐Chun Wu, Pin‐Chi Liao, Sung‐Chieh Chiu, Chia‐Wen Lu, Christos Panagopoulos, Atsushi Fujimori, Jenq‐Shinn Wu, Chi‐Te Liang, Sheng‐Di Lin, Shun‐Tsung Lo

**Affiliations:** ^1^ Institute of Electronics National Yang Ming Chiao Tung University Hsinchu Taiwan; ^2^ Department of Electrophysics National Yang Ming Chiao Tung University Hsinchu Taiwan; ^3^ Department of Physics National Taiwan University Taipei Taiwan; ^4^ Division of Physics and Applied Physics School of Physical and Mathematical Sciences Nanyang Technological University Singapore Singapore; ^5^ Department of Physics University of Tokyo Tokyo Japan; ^6^ Department of Physics and Center for Quantum Science and Technology National Tsing Hua University Hsinchu Taiwan; ^7^ Department of Electronic Engineering National Changhua University of Education Changhua Taiwan; ^8^ Center For Emergent Functional Matter Science National Yang Ming Chiao Tung University Hsinchu Taiwan

**Keywords:** epitaxial aluminum on miscut substrates, polycrystalline aluminum, single‐crystal aluminum, superconductivity modulation, twinned aluminum

## Abstract

Aluminum (Al) has attracted considerable attention for uses in photonic, electronic, and quantum devices. Its grain architecture governs surface roughness, electron and light scattering, and quantum decoherence, all of which critically affect device performance. Enhancing crystalline domain size and refining granularity control remain an ongoing research focus for producing ultraclean nanofilms. This study investigates the crystallinity of epitaxial Al grown on miscut GaAs substrates and examines its influence on Al superconductivity. The introduction of a substrate miscut alters Al growth kinetics, enabling the formation of twinned grains, polycrystalline structures, and micrometer‐scale single crystal. Variations in grain architecture result in approximately 10%, 100%, and 1000% modulation of the superconducting critical temperature, current, and magnetic field, respectively, while maintaining constant channel geometries. Reducing macroscopic grain boundaries decreases the Al nanofilm resistivity but enhances strain‐induced crystallinity deterioration, driving a transition from type‐I to type‐II‐like superconducting behavior. We suggest that preparing Al nanofilms, which approach an ultraclean limit in terms of surface quality, crystallinity, and transport properties, requires careful control of substrate miscut as well as the grain architecture. These findings highlight a tunable approach to controlling Al granularity and superconductivity via miscut, lattice‐mismatched substrates.

## Graphical abstract

The miscut substrate strategy offers a novel means to engineer aluminum surface roughness, grain architecture, crystallinity, and both normal and superconducting transport properties toward their clean limits by precisely tuning the miscut angle without altering growth conditions. Furthermore, the lattice mismatch strain induced by a substrate miscut can drive a transition from type‐I to type‐II‐like superconducting behavior.

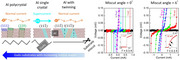

## 1 Introduction

The advancement of modern technologies is increasingly dictated by the ability to engineer materials at the nanoscale, where even subtle defects can profoundly alter performance. Achieving precise control over microstructures at this scale is not only a technical challenge but also a critical pathway toward unlocking new material functionalities [1, 2, 3, 4]. Aluminum (Al) thin films, in particular, have attracted significant attention for their critical roles in plasmonics and quantum computing. In the ultraviolet regime, Al acts as a plasmonic material essential for manipulating light, a capability vital to the development of advanced optical devices [5, 6, 7, 8, 9, 10]. In addition, Al‐based superconducting devices and circuits form the fundamental building blocks of superconducting qubits, the basic units of quantum computation [11, 12, 13, 14]. Crystal quality and grain architecture determine device performance: Al thin films with high crystallinity and atomically smooth surfaces enable plasmonic nanolasers to operate at room temperature with low threshold pumping power [5, 6, 7, 8, 9, 10], while also enhancing the coherence and stability of superconducting qubits [11, 12, 13, 14, 15, 16, 17, 18, 19]. These findings underscore the significance of precise control over the atomic structure of Al for advanced device applications.

Despite the growing demand for high‐quality Al thin films, their production remains challenging. Achieving wafer‐scale, atomically smooth films with excellent crystallinity is difficult, particularly due to the reactivity of aluminum with residual gases in the growth chamber, which can introduce unwanted impurities and defects. Molecular beam epitaxy (MBE) has emerged as an ideal technique for growing epitaxial Al films, offering atomic‐scale precision in controlling film thickness and composition. High‐quality epitaxial Al (111) thin films, ranging in thickness from the nanoscale to the atomic scale, have been successfully grown on various substrates, including silicon (Si), sapphire, and gallium arsenide (GaAs)—each offering distinct advantages for specific applications [18, 19, 20, 21, 22, 23]. However, a persistent challenge is the formation of grain boundaries. Even with MBE, twin grain boundaries in Al films are unavoidable. In face‐centered cubic (FCC) materials like Al, twins are planar defects that can significantly alter material properties [24, 25, 26, 27, 28, 29, 30, 31, 32]. These defects scatter electrons, thus degrading electrical conductivity, and introduce energy levels within the band gap (if it exists), which can undermine the thermal and mechanical stability of the films and ultimately limit their performance in demanding applications. Twin boundary formation can arise from several mechanisms, including lattice mismatch between the Al film and substrate [30, 31], preferential growth of certain crystal orientations on differently terminated substrate surfaces, and stacking faults in the crystal structure [28, 29]. For example, in an Al (111) thin film grown on a GaAs (001) substrate, the (111)‐oriented Al has a three‐fold rotational symmetry along the [111] direction, whereas the (001)‐oriented GaAs exhibits a two‐fold rotational symmetry. This mismatch in crystal symmetry promotes twin formation in Al films [21].

This study explores the use of GaAs substrates with varying miscut angles to suppress twin formation and control grain architecture in Al, aiming for the extreme clean limit. On miscut GaAs surfaces, vicinal step‐terrace patterns align with the miscut direction. They provide nucleation sites for Al adatoms and promote the growth of a single preferred crystalline domain, thereby minimizing twin formation. We employ vicinal GaAs (001) substrates with varying miscut angles of 0°, 2°, 6°, and 15° to modulate Al (111) nucleation, enable orientation‐selective epitaxial growth, and control Al twinning and crystallinity at the atomic scale. The surface morphology, crystallinity, and microstructure of the 40‐nm‐thick Al films were examined using atomic force microscopy (AFM), X‐ray diffraction (XRD), transmission electron microscopy (TEM), and scanning transmission electron microscopy (STEM). Twin Al crystalline domains are present on GaAs substrates with 0° and 2° miscuts but are largely suppressed at 6°, producing micrometer‐scale single‐crystal Al. As the miscut angle increases to 15°, a new Al (220) domain emerges. To examine how granularity influences electrical properties, we performed transport and magnetotransport measurements on Hall‐bar‐patterned Al nanofilms in both the normal and superconducting states. Notably, the twin‐suppressed, single‐crystal Al nanofilm on the 6°‐miscut substrate exhibits reduced charge‐defect scattering, enhanced superconductivity, and a transition to type‐II‐like superconducting behavior, which are associated with the reconstruction of atomic‐scale lattice mismatch strain from being localized near grain boundaries to being broadly distributed across the entire crystal. These results underscore the potential of substrate miscut‐driven domain engineering as a powerful strategy to tailor the electrical properties of Al and related materials for advanced device applications.

## 2 Results and Discussion

**FIGURE 1:**
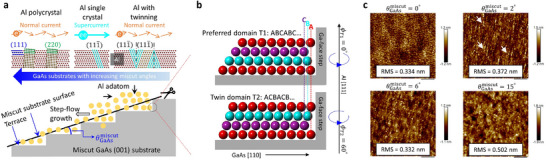
Growth mechanism and surface characterization of epitaxial Al on miscut GaAs substrates. (a) Schematic diagrams (top) of three distinct epitaxial Al atomic structures induced by vicinal GaAs (001) substrates with different miscut angles $\theta_{\mathrm{GaAs}}^{\mathrm{miscut}}$ (bottom). Lines of varying orientation denote different lattice planes. The substrate miscut generates a surface‐step‐terrace structure that facilitates Al step‐flow growth. A schematic diagram regarding two distinct conducting current pathways in different grain architectures is also presented. (b) Stacking sequences of Al twin crystalline domains, where T1 and T2 correspond to ABCABC… and ACBACB… arrangements, respectively, with the two domains related by a 60° rotation about the [111] direction. The letters A, B, and C denote three distinct atomic positions, located closest to, midway from, and furthest from the Ga‐face step. The step terraces act as nucleation sites that favor the growth of ABCABC stacking sequence. (c) Surface morphology images of 40‐nm‐thick Al films grown on GaAs substrates with $\theta_{\mathrm{GaAs}}^{\mathrm{miscut}}=$ 0°, 2°, 6°, and 15°, obtained by atomic force microscopy. The root‐mean‐square (RMS) roughness is calculated for each sample. The arrows mark the Al line structures formed to conform to the surface terrace steps of the GaAs substrate. The scale bar for all four images is 1 µm.

Figure 1a shows a schematic illustrating the epitaxial growth of Al nanofilms with controllable grain architectures and electrical properties on commercially available, miscut GaAs substrates studied in this work. In our work, all the substrates were thermally desorbed to remove the native oxide and then capped with an MBE‐grown Ga‐terminated GaAs layer, yielding an atomically clean surface with step terraces defined by vicinal lattice planes and formed without any additional etching. Because the GaAs substrates are kept at low temperatures (<0°C; beyond the range of the thermometer) during Al deposition, the step terrace structure remains intact and atomic interdiffusion is suppressed. The step terraces are deliberately introduced to guide step‐flow Al lateral growth via step‐edge Al‐Ga bonding. Although ideally the step height remains nearly constant (≈3 Å), the terrace width varies with the miscut angle of the substrate (see Figure S1). The widths are approximately 80 and 24 Å for the 2° and 6° miscut substrates, respectively. For the 15° miscut, two terrace widths of roughly 8 and 12 Å are expected. During the epitaxy of Al nanofilms, Al islands first nucleate within each terrace and, as the growth proceeds, expand and coalesce into a continuous film across the terraces, in contrast to the layer‐by‐layer growth mode on flat substrates [33]. In order to ensure a pristine Al/GaAs interface, all processes were performed in situ without air exposure (see the Experimental Section for details). For the relatively thick 40‐nm Al films studied here, the Al crystalline structure, which is determined by nucleation and dislocation behavior at the Al/GaAs interface, dominates the transport properties. By tuning the substrate miscut angle, we systematically control the crystalline domains and twin formation in the Al films. Variations in terrace width (governing domain matching) and terrace number (setting preferential nucleation sites) enable the formation of single‐crystal, or twinned, or polycrystalline Al. This approach is physically similar to the previously reported two‐step method. In their study, high‐quality silver seed islands are formed prior to the growth of a continuous film but the underlying mechanisms are entirely different from ours [33]. In our case, the Al growth conditions remain unchanged, whereas their method requires adjusting the growth parameters between the two steps.

Figure 1b demonstrates the reduction of twin domains in Al nanofilms grown on a miscut GaAs substrate. Along the [111] direction, two Al domains with a 60° rotational symmetry can form: one with an ABCABC… stacking sequence and the other with an ACBACB… sequence. To encourage orientation‐selective growth, the vicinal substrate was prepared with a Ga‐rich surface. Epitaxial growth proceeds through a step‐flow mode, guided by Al‐Ga bonding along the step sidewalls. The bonding energetics and therefore the bonding strength are governed by the lateral Al‐Ga separation. Shorter distances between Al adatoms and the step edge correspond to lower‐energy configurations [30]. Among the three atomic sites, position A lies closest to the Ga‐face step, followed by B and then C. Due to the stronger Al‐Ga bonding, Al adatoms in the first layer preferentially occupy site A. In the second layer, they favor site B over C, promoting the formation of the T1 domain (ABCABC… stacking) and suppressing the T2 domain (ACBACB… stacking). Figure 1c shows the surface morphology of 40‐nm‐thick Al films grown on GaAs substrates with miscut angle of 0° (sample A), 2° (sample B), 6° (sample C), and 15° (sample D). Samples A and C exhibit the smoothest surface with root‐mean‐square (RMS) roughness around 0.33 nm, whereas sample D shows the highest roughness at 0.50 nm. This variation stems from differences in Al crystallization and grain architecture governed by the substrate miscut angle. The Al surface step terrace structure from the vicinal GaAs substrate becomes evident at the 2° miscut because it can provide moderate twin suppression, Al/GaAs lattice misalignment, and crystallinity (Figure 1c, arrows). This substrate‐induced morphology is even more pronounced when the Al film thickness is reduced to 3.5 nm (Figure S7a). The AFM image, which is obtained after etching away the Al film, clearly reveals the GaAs surface step terrace structure formed by the various treatments before the Al growth (Figure S2). In addition, irregular GaAs step‐terrace bunching and Al island formation increasingly affect the Al surface roughness as the miscut angle rises [34]. The detailed morphology also correlates closely with the Al grain structure. We will next analyze these grain structures and investigate how these impact both normal and superconducting charge transport. In this work, relatively thick nanofilms are employed to minimize the effects of surface scattering, quantum confinement, and even 2D superconductivity, thereby enabling a clearer elucidation of the substrate miscut influence.

**FIGURE 2:**
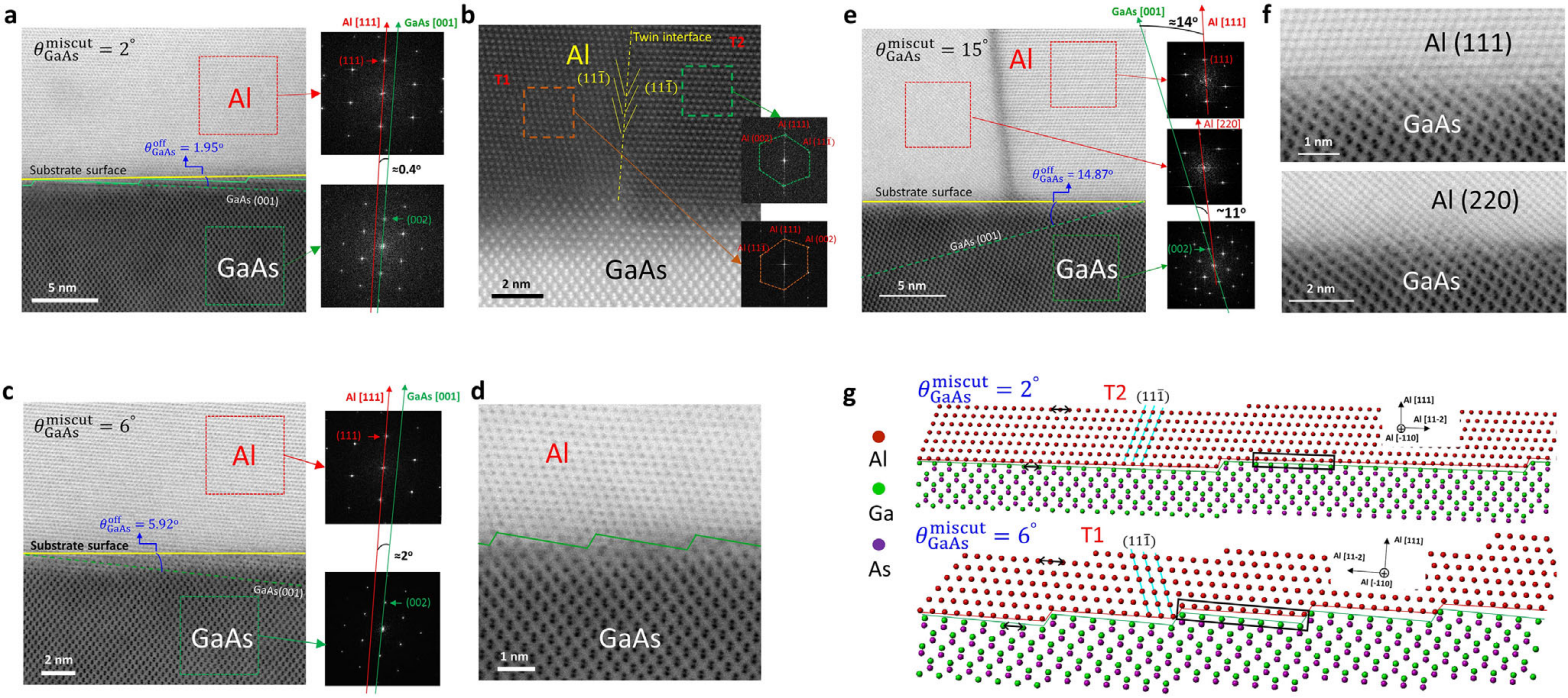
Atomic structures of epitaxial Al crystalline domains on miscut GaAs substrates. (a) Annular bright‐field (ABF) scanning transmission electron microscopy (STEM) image and corresponding fast Fourier transform (FFT) patterns of a 40‐nm‐thick Al film grown on a 2°‐miscut GaAs (001) substrate (sample B). A step‐terrace structure (outlined in green) is observed along the vicinal substrate surface (outlined in yellow). The dashed line marks the GaAs (001) lattice plane. The off angles of the GaAs (001) and Al (111) lattice planes relative to the substrate surface ($\theta_{\mathrm{GaAs}}^{\mathrm{off}}$ and $\theta_{\mathrm{Al}}^{\mathrm{off}}$, respectively) are controlled via the substrate miscut. They are experimentally determined through X‐ray diffraction (XRD) measurements (Figure S4 and Table S1). The angle between GaAs (001) and Al (111) lattice planes, that is, $\theta_{\mathrm{GaAs}}^{\mathrm{off}}-\theta_{\mathrm{Al}}^{\mathrm{off}}$, is also obtained by comparing their STEM‐FFT patterns. (b) High‐angle annular dark‐field (HAADF) STEM image showing a twin interface between T1 and T2 domains in sample B. (c, d) ABF‐STEM image of a 40‐nm‐thick Al film grown on a 6°‐miscut GaAs substrate (sample C), illustrating: (c) the orientation relationship between the GaAs (001) lattice plane and substrate surface; (d) a series of atomically flat terraces at the Al/GaAs interface. (e,f) ABF‐STEM image of a 40‐nm‐thick Al film grown on a 15°‐miscut GaAs substrate (sample D), illustrating: (e) the two different crystalline domains with corresponding FFT patterns; (f) the Al (111)/GaAs (001) and Al (220)/GaAs (001) interfaces. Note that the $\theta_{\mathrm{GaAs}}^{\mathrm{off}}$ values shown in (a), (c), and (e) are from XRD analysis (Table S1). (g) Schematic models illustrating the atomic arrangement of Al on the surface step terrace of 2°‐miscut and 6°‐miscut GaAs (001) vicinal substrates. The misalignment between Al (111) and GaAs (001) lattice planes becomes larger at the 6° substrate miscut. The double‐headed arrows denote the spacing of the Al {112} or Ga {110} planes while the rectangles denote the domain matching units.

High‐resolution STEM analyses were carried out to explore the microstructure of epitaxial Al nanofilms on GaAs substrates with varying miscut angles. As shown in Figure 2a, the annular bright‐field (ABF) STEM image of Al/2°‐miscut GaAs heterostructure clearly demonstrates the orientation relationship among Al (111), GaAs (001), and the substrate surface, which is in good agreement with the XRD results (Figure S4 and Table S1, which show the measured off angles $\theta_{\mathrm{GaAs}}^{\mathrm{off}}$ and $\theta_{\mathrm{Al}}^{\mathrm{off}}$ of the GaAs and Al lattice planes relative to the substrate surface, discussed latter in this paper). Fast Fourier transform (FFT) analysis reveals a small ≈0.4° misalignment between the Al (111) and GaAs (002) lattice planes, consistent with the XRD $\theta_{\mathrm{GaAs}}^{\mathrm{off}}-\theta_{\mathrm{Al}}^{\mathrm{off}}$ results (Table S1). This indicates that the Al (111) crystal is closely aligned with the GaAs (001) substrate when the miscut angle is as small as 2°. The cross‐sectional high‐angle annular dark‐field (HAADF) STEM and corresponding FFT pattern reveal the twin domain structure and interface (Figure 2b; see Figure S3 for a zoom‐in view highlighting the clean twin interface).

When the miscut angle reaches 6°, the misalignment between Al (111) and GaAs (001) increases to ≈2° (Figure 2c), similarly consistent with the $\theta_{\mathrm{GaAs}}^{\mathrm{off}}-\theta_{\mathrm{Al}}^{\mathrm{off}}$ values obtained from XRD measurements (Figure S4 and Table S1). The high‐resolution ABF‐STEM image in Figure 2d reveals the vicinal Al/GaAs interface and atomically flat terraces. When the miscut angle increases up to 15°, a new Al (220) domain emerges, as shown in the ABF‐STEM image and correspond FFT patterns (Figure 2e), with no indication of twin structures. The Al (111) and Al (220) domains are misaligned with the GaAs (001) substrate by approximately 14° and 11°, respectively, again consistent with the XRD $\theta_{\mathrm{GaAs}}^{\mathrm{off}}-\theta_{\mathrm{Al}}^{\mathrm{off}}$ results (Figure S4 and Table S1). A sharp interface is observed between the Al (111) and Al (220) domains. High‐resolution ABF‐STEM images in Figure 2f further resolve the Al (111)/GaAs (001) and Al (220)/GaAs (001) interfaces. Collectively, these results demonstrate clean Al/GaAs interfaces, clean twin grain boundaries, and clean polycrystalline grain boundaries, all without AlO_x_ formation. Figure 2g illustrates the atomic arrangement of Al on the step terraces of a vicinal GaAs (001) substrate, highlighting the epitaxial growth of Al on GaAs via domain matching epitaxy (DME) [33, 34, 35]. The lattice matching between the Al {112} and Ga {110} planes (with separations of 4.96 and 4.00 Å, respectively) is strongly affected by the miscut angle of the GaAs substrate miscut. The Ga‐rich GaAs surface, which is not ideally lattice‐matched with Al, introduces strain on the initially grown Al layers. Within each domain matching region, this strain can be locally relaxed. On the other hand, residual domain mismatching and misfit strain are further relieved through a slight periodic modulation of domain size.

We note that for the Al nanofilm grown on a flat (miscut angle = 0) GaAs substrate, the optimal DME condition occurs when four {112} spacings of Al along the [11‐2] direction (≈4 × 4.96  =  19.84 Å) align with five {110} spacings of Ga (≈5 × 4.00  =  20.00 Å). This configuration leaves a rather small residual in‐plane tensile strain of −0.16 Å, which can be relaxed through multiple domain matching processes. When the substrate miscut angle is increased to 2°, the terrace width corresponds to approximately twenty {110} Ga spacings (≈80 Å), whereas at 6°, it narrows to about six {110} Ga spacings (≈24 Å). As presented in the upper panel of Figure 2g for the case of 2° miscut, 4/5 domain matching remains effective despite a small misalignment angle of approximately 0.4° between the Al film and GaAs substrate (indicated by the rectangle). In contrast, for the situation of 6° miscut (lower panel), the matching condition shifts to five {112} Al spacings against six {110} Ga spacings (i.e., 5/6 domain matching) as a result of a larger Al (111)/GaAs (001) misalignment and narrower terrace widths (also highlighted by the rectangle). This causes an increased lattice mismatch of 0.80 Å and a stronger compressive strain compared with that of the Al/2°‐miscut GaAs sample. We note that beyond the DME modulation, a larger miscut angle also increases the number of nucleation sites, thereby suppressing twin formation and allowing strain effects to persist in thicker Al films rather than relaxing at grain boundaries. Moreover, mismatch between neighboring atomic‐scale Al islands across adjacent terraces introduces additional strain, which is required to form a continuous Al film. This can account for the observed misalignment between the Al (111) planes and the GaAs substrate. Consequently, it is expected that the 40‐nm‐thick Al film experiences significant lattice deformation from the complex strain interplay between the Al/GaAs lattice mismatch and Al domain mismatch [34].

**FIGURE 3:**
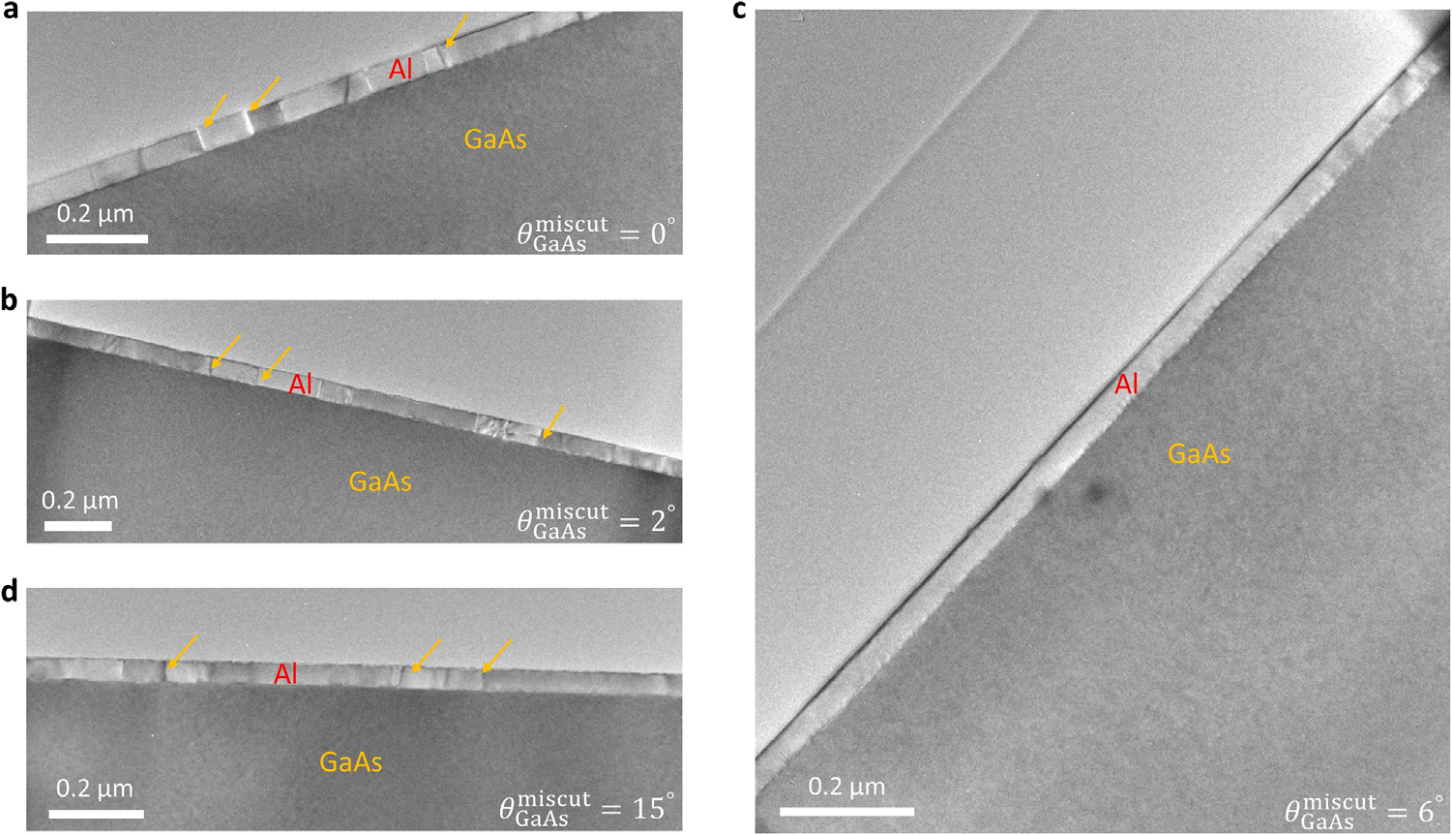
Al twinning suppression via miscut GaAs substrates. (a–d) Low magnification cross‐sectional TEM images of 40‐nm‐thick Al films grown on GaAs substrates with miscut angles of (a) 0°, (b) 2°, (c) 6°, and (d) 15°. The arrows mark the grain boundaries.

The influence of GaAs substrates with varying miscut angles on the suppression of twin formation in epitaxial Al nanofilms is also evident in the low‐magnification cross‐sectional TEM images (Figure 3). It is evident that Al nanofilms grown on nominally flat (0° miscut) and 15°‐miscut GaAs substrate exhibit a high density of domain boundaries (Figure 3a,d). In contrast, the film on the 2°‐miscut substrate shows a noticeably reduced domain density (Figure 3b). Remarkably, the Al/6°‐miscut GaAs sample displays a complete absence of boundaries over the observed range of ≈1.6 µm (Figure 3c). This observation aligns with the XRD azimuthal scan results discussed later, confirming that a 6° substrate miscut yields substantial twin suppression.

**FIGURE 4:**
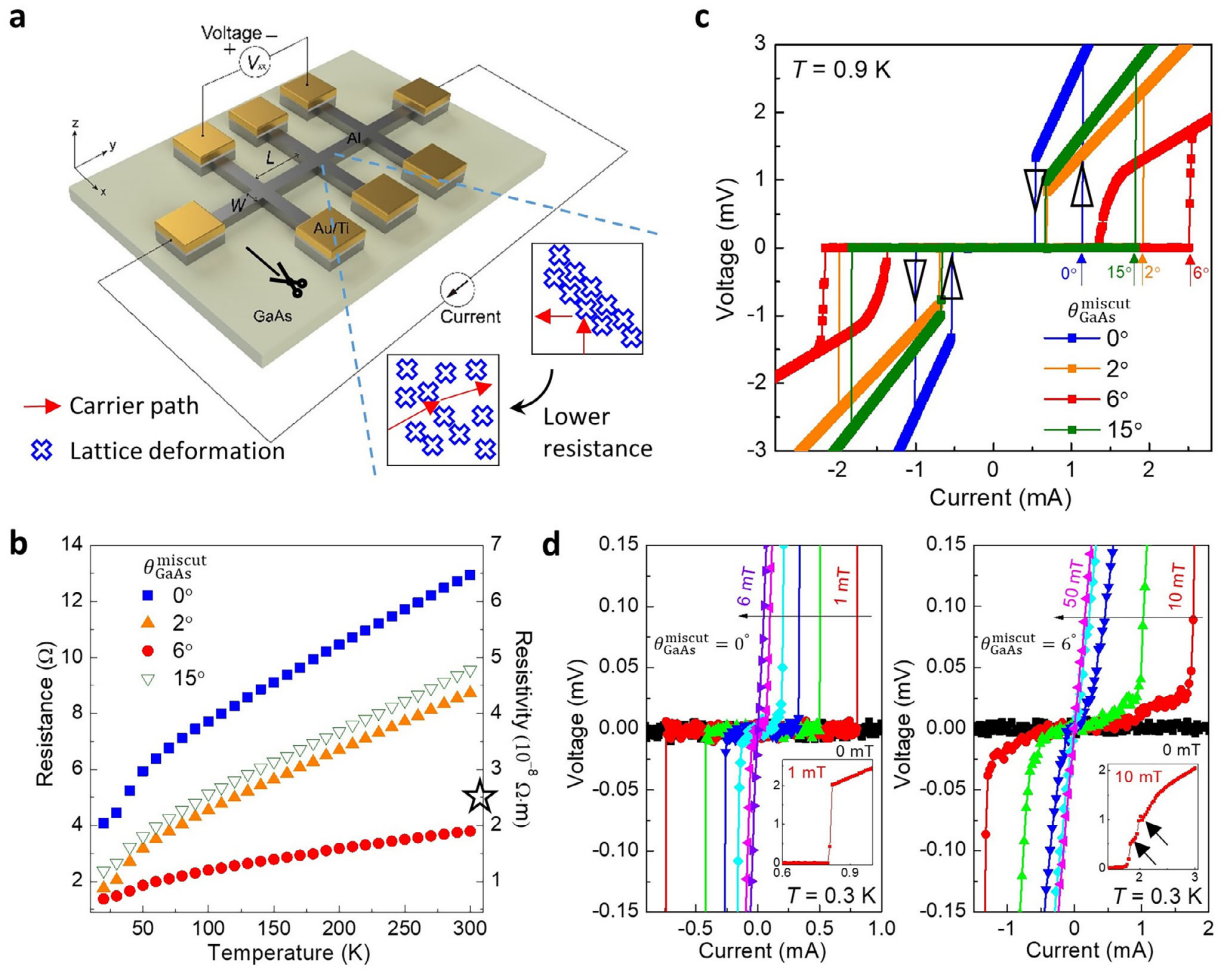
Al superconductivity modulation via miscut GaAs substrates. (a) Schematic of the studied Al Hall‐bar devices with a channel length‐to‐width ratio *L*/*W* = 8 for four‐terminal transport and magnetotransport measurements. The bold line marks the [110] miscut direction, which is perpendicular to the Hall‐bar channel. Insets, Al lattice deformation regions and electron scattering paths predicted for the Al/0°‐miscut GaAs (top) and Al/6°‐miscut GaAs (bottom) samples. (b) Four‐terminal resistance and the corresponding resistivity as a function of temperature for four Al/miscut GaAs samples. The star denotes the predicted resistivity value for bulk Al. (c) Four‐terminal voltage‐current responses under forward and backward current sweeps (indicated by upward and downward open arrowheads, respectively) for four Al/miscut GaAs samples. The positive critical currents during the forward sweep are indicated by arrows. (d) Four‐terminal voltage‐current responses during forward current sweeps under various magnetic fields for the Al/0°‐miscut GaAs and Al/6°‐miscut GaAs samples. Insets, same data highlighting the transition between the superconducting and normal states, with the arrow indicating a step‐like feature.

As shown in Figure S4, XRD 2*θ*‐*θ* scans of the four samples reveal the Al (111) and Al (222) reflections, where *θ* (2*θ*) is the angle between the incident X‐ray beam and lattice plane (detector). The XRD peaks appear when Bragg reflections occur from the lattice. It is important to note that the 2*θ*‐*θ* signals were calibrated with respect to the Al (111) lattice plane, while the GaAs (00*l*) substrate signal gradually weakens and eventually disappears as the miscut angle increases. This suggests that the Al (111) lattice plane becomes misaligned with the GaAs (001) substrate, in qualitative agreement with the STEM observations. Increasing the miscut angle from $\theta_{\mathrm{GaAs}}^{\mathrm{miscut}}=$ 0°–2° leads to higher Al (111) diffraction peaks, reflecting improved Al atomic ordering. However, further increases in the miscut angle lead to a steady decrease in peak intensity, signifying reduced crystallinity. Although the 40‐nm‐thick Al film on the GaAs substrate with a miscut angle of 6° attains a grain‐boundary‐free clean limit, this comes at the expense of its crystallinity, which is partially degraded to relax the lattice mismatch strain between Al and GaAs. This trend is also evident in the rocking curves obtained from *ω* scans, where *ω* is the angle between the incident X‐ray beam and substrate surface (Figure S5a). The full width at half maximum (FWHM) of the Al (111) reflections increases with substrate miscut angle, measured at approximately 0.17°, 0.11°, 1.35°, and 3.70° for the $\theta_{\mathrm{GaAs}}^{\mathrm{miscut}}=$ 0°, 2°, 6°, and 15° samples, respectively. The off angles $\theta_{\mathrm{Al}}^{\mathrm{off}}$ between the Al (111) plane and substrate surface and $\theta_{\mathrm{GaAs}}^{\mathrm{off}}$ between the Ga (001) plane and substrate surface are calculated from the rocking curve as |ω − θ| with *θ* being the Bragg diffraction angle for the corresponding lattice plane. The obtained $\theta_{\mathrm{GaAs}}^{\mathrm{off}}$ values also confirm the intended miscut angles of the GaAs (001) substrates. The misalignment between the Al (111) and GaAs (001) planes can be determined from the difference between $\theta_{\mathrm{Al}}^{\mathrm{off}}$ and $\theta_{\mathrm{GaAs}}^{\mathrm{off}}$, yielding 0.40° and 2.23° for the cases of miscut angles of 2° and 6°, respectively. For the Al grown on substrate with a miscut angle of 15°, two different values of 14.27° and 10.87° are obtained for the Al (111) and Al (220) planes. These results are in quantitative agreement with those derived from the STEM data (Figure 2). All measured and derived angular data are collected in Table S1 and schematically illustrated in the insets of Figure S4. In order to evaluate the effect of substrate miscut on twin suppression, X‐ray azimuthal *ϕ*‐scans were carried out for Al (200) as shown in Figure S6. For Al nanofilms grown on the flat GaAs substrate, both XRD peaks from T1 and T2 twin domains are clear observed [36]. The intensity of Al (200) peaks associated with the T2 domain decreases at a miscut angle of 2° and nearly vanishes at 6°. This *ϕ*‐scan observations suggest that twin suppression enhances surface flatness, which consistently leads to an increased Al/GaAs misalignment angle extracted from the 2*θ*‐*θ* scans. We note that for the case of the 6° miscut complete twin suppression and optimal surface smoothness can be achieved, but at the expense of crystallinity. On the other hand, the 2° miscut provides the best crystallinity but results in a higher density of twin boundaries and increased surface roughness. At a miscut angle of 15°, the T2 contribution disappears entirely, but a new domain emerges (indicated by green arrows in Figure S6). This new domain is identified as Al (220) with an off angle of $\theta_{\mathrm{Al}}^{\mathrm{off}}\approx$ 4°, as illustrated in the insets of Figure S4d. These XRD results demonstrate that twin formation in Al nanofilms can be effectively suppressed by using miscut GaAs substrates, with a critical miscut angle of approximately 6°. However, when the miscut angle exceeds 6°, even at the expense of increased lattice deformation, the Al nanofilm can no longer maintain a single‐crystal structure. The intensified Al/GaAs lattice mismatch strain, which originates from the reduced terrace width and constrained domain matching regions, facilitates the emergence of Al (220) domains and grain boundaries as a means of strain relaxation. This ultimately pushes the film toward polycrystallinity. Interestingly, the intermediate miscut angle (2°) achieves an optimal balance between step‐edge nucleation density and domain matching distance, resulting in the highest crystallinity observed in this study. Nonetheless, this enhanced crystallinity does not translate into the smoothest Al surface (Figure 1c) or the lowest normal‐state resistivity (Figure 4b). These experimental results can be ascribed to the relatively large terrace width that still accommodates non‐negligible twin boundaries.

Figure 4a presents a schematic of an Al/miscut GaAs Hall‐bar device configured for four‐terminal measurements to examine the electrical properties of Al in both its normal and superconducting states. As illustrated in Figure 4b, increasing the GaAs substrate miscut angle to 6° leads to resistance minima across the entire temperature range. However, further increasing the miscut up to 15° results in higher resistance, attributed to the emergence of a polycrystalline structure. This comparison indicates that charge‐defect scattering exerts the least influence on normal‐state current conduction in the twin‐suppressed, single‐crystal Al grown on the substrate with a miscut angle of 6°, which shows a room‐temperature resistivity (≈1.9 × 10^−8^ Ω·m) lower than that of bulk Al (≈2.7 × 10^−8^ Ω·m; Figure 4b, star) [37]. The corresponding values are 6.5 × 10^−8^ Ω·m, 4.4 × 10^−8^ Ω·m, and 4.8 × 10^−8^ Ω·m for the miscut angles of 0°, 2°, and 15°, respectively. A quantitative comparison of the room‐temperature resistivity across different miscut substrates and film thicknesses will be further discussed in Figure 6. Low‐magnification cross‐sectional TEM images confirm that macroscopic grain boundaries are suppressed up to a 6° miscut but reappear at 15° (Figure 3). Additionally, STEM imaging reveals clean homointerfaces between adjacent Al grains, formed with lattice deformation but without the presence of AlO_x_ (Figure 2 and Figure S3). These findings link the variation in resistance with the degree of charge‐defect scattering, which is influenced by the strength and distribution of lattice deformation. The Al film on the flat GaAs substrate (miscut angle = 0°) has many grain boundaries that localize strain and cause stronger electron scattering. In contrast, the 40‐nm‐thick film grown on the substrate with a 6° miscut angle has strain distributed across a single crystal, resulting in longer electron mean‐free‐path and hence lower resistivity (Figure 4a, inset). The strain effect can modulate the band structure and consequently may reduce the electron effective mass, resulting in an additional decrease in resistivity (Figure 4b).

Figure 4c shows the superconducting voltage‐current characteristics with hysteresis phenomena, which are obtained from both forward and backward current sweeps. As the current magnitude increases, superconductivity, which is characterized by zero voltage drop, is disrupted at distinct critical current values for each Al/miscut GaAs sample (marked by solid arrows). The observed hysteresis may arise from multiple mechanisms, including vortex pinning in type‐II superconducting films [38, 39], phase slips in superconducting wires [40, 41, 42, 43], and electron heating [43, 44]. Here, we present the data taken at 0.9 K, where a lower critical current and reduced Joule heating effect allow us to observe reproducible hysteresis behavior. However, their individual contributions cannot be separately identified in our measurement setup with continuous current sweeps and varying current ranges used for different samples. Although the critical current differs between sweep directions, both follow a consistent trend with the miscut angle, reaching maxima at a 6° substrate miscut. Notably, the modulation ratio of the positive critical current during the forward sweep, defined as (*I*_c_(6°) − *I*_c_(0°))/*I*_c_(0°), reaches up to 120%. A significantly higher ratio is expected at lower temperatures. At a 6° substrate miscut, the critical current rises while the normal‐state resistance decreases compared with the 0° miscut. Intriguingly, this enhancement is associated with a broadened current range between the superconducting and normal‐metal states. This behavior stands in contrast to the conventional expectation for type‐I superconductors, in which cleaner systems, which are characterized by lower resistivity and greater coherence, generally exhibit sharper superconducting transitions. The TEM and XRD analyses provide clues to the observed link between superconducting and normal charge transport properties.

Cross‐sectional TEM data reveal that macroscopic grain boundaries are largely eliminated in Al nanofilms grown on the 6°‐miscut substrate, accompanied by a reduction in resistance. However, both XRD 2*θ*‐*θ* and *ω* scans indicate degraded crystallinity in Al on the 6°‐miscut substrate, despite its lower resistance compared to the 0°‐ and 2°‐miscut samples. This comparison indicates that charge‐defect scattering at grain boundaries predominantly governs the normal‐state resistance, while a separate scattering mechanism likely influences superconductivity with minimal impact on normal‐state properties. Grain boundaries concentrate lattice deformation and relieve stress from the Al/GaAs lattice mismatch, enabling each Al grain to retain high crystallinity. When these boundaries are absent, the effect of mismatch strain becomes distributed throughout the crystal, leading to reduced overall crystallinity, as observed in the film grown on the 6°‐miscut substrate. The resulting widespread distribution of lattice deformation leads to small‐angle scattering that minimally affects the Drude resistance but can induce local quantum decoherence (Figure 4a, bottom insets).

**FIGURE 5:**
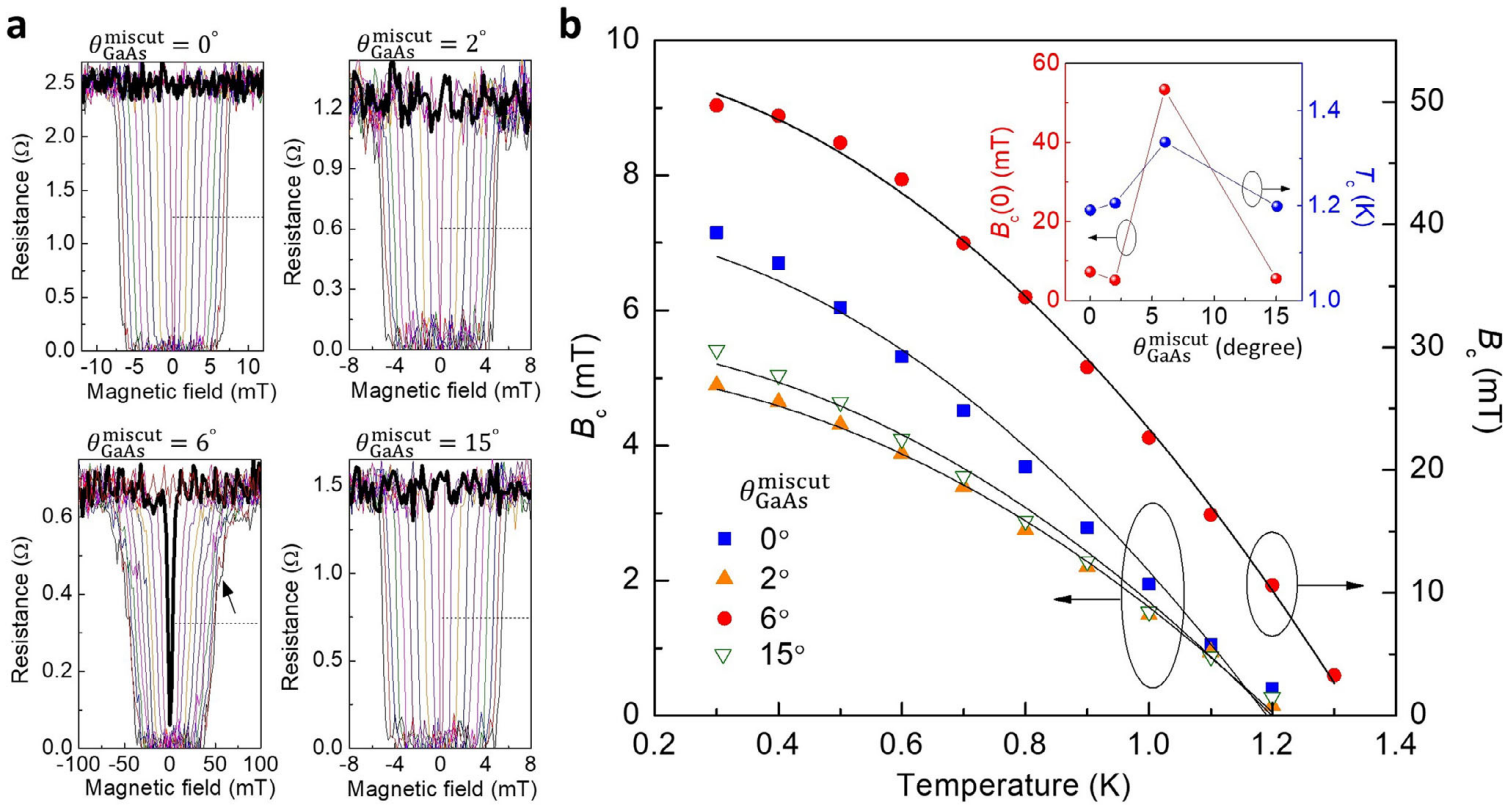
Al superconductivity characteristics at different substrate miscut angles. (a) Four‐terminal resistance as a function of magnetic field for four Al/miscut GaAs samples with varying the temperature from *T* = 0.3–1.3 K (extended to 1.4 K for the $\theta_{\mathrm{GaAs}}^{\mathrm{miscut}}=$ 6° sample). The critical magnetic field is defined as the field at which the resistance drops to half of its normal‐state value (indicated by dashed lines). The bold traces indicate the data at *T* = 1.3 K. The arrow indicates the step‐like structure during the superconducting transition. (b) Temperature dependence of the critical magnetic field *B*_c_ for four different $\theta_{\mathrm{GaAs}}^{\mathrm{miscut}}$ values. Inset, zero‐temperature critical field *B*_c_(0) and critical temperature *T*_c_ as a function of $\theta_{\mathrm{GaAs}}^{\mathrm{miscut}}$.

Figure 4d shows the superconducting voltage‐current characteristics under a varying out‐of‐plane magnetic field, which similarly destroys superconductivity. The Al/0°‐miscut GaAs sample retains near‐zero resistance throughout the superconducting region and exhibits a sharp transition to the normal state regardless of the applied magnetic field (left panel, inset), consistent with typical type‐I superconductivity with macroscopic coherence. In contrast, the Al/6°‐miscut GaAs sample has finite current dissipation within the superconducting region and undergoes a gradual superconducting transition through multiple voltage steps in the presence of a magnetic field (right panel, inset). They are reminiscent of type‐II superconductors, where local penetration of magnetic flux forms vortices. According to the current understanding of type‐II superconductors, zero resistance is maintained when vortices are pinned at defects. Once vortices begin to move, finite resistance appears, and sequential vortex depinning produces step‐like features during superconducting breakdown, as shown in Figures 4d and 5a [39, 45, 46, 47, 48, 49, 50, 51, 52, 53, 54, 55]. We believe that our Al‐based superconducting system, featuring a controllable microstructure, provides valuable insight into the behavior of unconventional superconductors, which usually exhibit type‐II characteristics. Moreover, our experimental results may stimulate further studies involving direct microscopic visualization of vortex distributions and dynamics using scanning tunneling microscopy or superconducting quantum interference device (SQUID) magnetometry techniques [55, 56]. The single‐crystal Al nanofilm grown on the GaAs substrate with a 6° miscut angle maintains zero resistance up to higher currents and magnetic fields, whereas films with more grain boundaries enter dissipative states at lower thresholds. Consequently, as shown in Figure 4, the critical current modulation ratio can be much higher than 100% in an applied magnetic field.

We observe that superconductivity persists to higher magnetic fields in the current‐voltage relations for the $\theta_{\mathrm{GaAs}}^{\mathrm{miscut}}=$ 6° sample compared to the 0° sample. To quantify this enhancement, we examine the resistance as a function of the out‐of‐plane magnetic field, where resistance is calculated by dividing the measured voltage by the applied current (100 µA for the $\theta_{\mathrm{GaAs}}^{\mathrm{miscut}}=$ 6° sample and 50 µA for the others). The data are presented in Figure 5a. When $\theta_{\mathrm{GaAs}}^{\mathrm{miscut}}=$ 6°, the superconducting transition proceeds over a broadened magnetic field range and exhibits step‐like structures with the magnetic field. This behavior is consistent with type‐II superconductivity as also demonstrated in the current‐driven transition. Moreover, we observe that an abrupt change in the resistance due to superconducting breakdown appears up to the temperature of *T* = 1.4 K for the $\theta_{\mathrm{GaAs}}^{\mathrm{miscut}}=$ 6° sample, slightly higher than the other samples (bold traces). Figure 5b presents the temperature dependence of the critical magnetic field *B*_c_, defined as the field where the resistance reaches half of the normal‐state value for each sample (as indicated by the dashed lines in Figure 5a). The data show good agreement with the relation *B*_c_(*T*) = *B*_c_(0)(1‐(*T*/*T*_c_)^2^), a form either supported by or approximated from various theoretical models and commonly observed in both type‐I and type‐II superconducting systems [45, 46, 47]. The extracted zero‐temperature critical magnetic field *B*_c_(0) and critical temperature both reach their maximum values for the $\theta_{\mathrm{GaAs}}^{\mathrm{miscut}}=$ 6° sample. Among all samples, the largest modulation ratios are 935% for *B*_c_(0) and 12% for *T*_c_.

The applied current generates a self‐induced magnetic field that can destroy superconductivity once it exceeds the critical field, a phenomenon known as the Silsbee effect [57]. Additionally, increasing the current or magnetic field raises the kinetic energy of Cooper pairs, leading to orbital pair‐breaking [58]. In principle, improved crystallinity leads to a larger superconducting gap and higher critical temperature. This should enhance both the critical current and critical field. In disordered superconductors, however, the absence of complete macroscopic coherence can cause these two parameters to vary independently when disorder is tuned. For the $\theta_{\mathrm{GaAs}}^{\mathrm{miscut}}=$ 2° sample, partial suppression of twin boundaries (as indicated by XRD in Figure S6) improves crystallinity relative to the $\theta_{\mathrm{GaAs}}^{\mathrm{miscut}}=$ 0° sample (Figure S5), resulting in a higher critical current but a lower critical field. This suggests that reducing twin boundaries at moderate miscut angles promotes macroscopic coherence. In contrast, further increasing the miscut angle to 6° enhances both the critical current and critical field of superconducting Al, despite the decrease in crystallinity. These distinct dependencies on miscut angle indicate that different mechanisms are responsible for the superconductivity modulation.

Let us correlate the XRD results (Figure S5a), which capture the film crystallinity, with the type‐II‐like superconducting characteristics, with the dissipative charge transport within the superconducting state and step‐like transition features. This reveals that the observed superconductivity enhancement arises from a dual mechanism: reduced dissipative scattering through grain boundary suppression and enhanced magnetic flux penetration driven by lattice deformation. The hallmark of type‐II superconductivity will be further confirmed by the parameter calculations that follow [45, 46]. The coherence length is estimated using $\xi =\sqrt{\phi_{0}/\left(2\pi B_{c}\left(0\right)\right)}$ (where ϕ_0_ =  *h*/2*e* is the flux quantum), yielding ξ ≈ 213, 253, 79, and 244 nm for samples with $\theta_{\mathrm{GaAs}}^{\mathrm{miscut}}=$ 0°, 2°, 6°, and 15°, respectively. Using the Pippard's relation 1/ξ  =  1/ξ_0_ + 1/*l*, where ξ_0_ =  1.6 µ*m* is the intrinsic Bardeen–Cooper–Schrieffer (BCS) coherence length for Al, we estimate the mean free path *l* ≈ 246, 300, 83, and 287 nm for the corresponding samples. Here, 1/*l* represents the average number of scattering events per unit length, incorporating all scattering mechanisms that influence both Drude resistance and superconducting properties. Moreover, the penetration length λ can be estimated via $\lambda ∼\lambda_{L}\sqrt{\xi_{0}/l}$, where λ_L_ =  16 nm is the intrinsic London penetration depth for Al. This yields λ ≈ 41, 37, 70, and 38 nm for samples with $\theta_{\mathrm{GaAs}}^{\mathrm{miscut}}=$ 0°, 2°, 6°, and 15°, respectively [45, 59]. According to these parameters, we calculate the Ginzburg‐Landau parameter κ  =  λ/ξ and obtain values of 0.19, 0.15, 0.90, and 0.16 for the corresponding samples. Notably, the value κ  =  0.90 for Al on the 6°‐miscut substrate exceeds the theoretical threshold of $\kappa =1/\sqrt{2}$ that separates type‐I and type‐II superconductivity [45, 46], supporting our observation of type‐II‐like superconducting behavior in this sample. It should be noted that both λ and κ can further increase with reducing the film thickness and enhancing the surface scattering, because the scattering length scale will be constrained by the thickness *d*. As the film is thinned toward the 2D limit (*d* ≪ λ), its ability to expel magnetic fields significantly weakens, and the relevant length scale for magnetic field penetration is no longer the conventional penetration depth but the Pearl length, which exhibits a much stronger dependence on film thickness [45].

**FIGURE 6:**
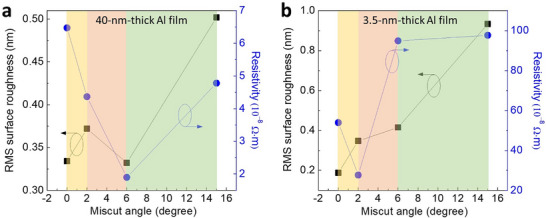
Interrelationship between surface roughness modulation and grain boundary control. Surface roughness (Figure 1c and Figure S7) and room‐temperature resistivity as a function of GaAs substrate miscut angle for (a) 40‐nm‐thick and (b) 3.5‐nm‐thick Al films, respectively. Shaded regions with varying colors correspond to different ranges of substrate miscut angles.

We have systematically studied the Al surface roughness, crystallinity, as well as both normal‐state and superconducting electrical properties, and demonstrated that achieving an ideal “clean” condition such as a smooth surface with minimized roughness, a perfect crystal with suppressed twin boundaries, a pure conductor with low resistivity, and a clean superconductor with minimal vortex density cannot be experimentally realized simultaneously using a single substrate miscut. In order to gain a comprehensive understanding of Al step‐flow growth on miscut substrates, which is a key knob governing grain architecture and consequently both normal and superconducting transport, we compare the Al surface roughness and normal‐state resistivity of 40‐nm‐thick and 3.5‐nm‐thick films (Figure 6 and Figure S7a). Domain matching epitaxy initiates within individual terraces, forming Al islands that enlarge and coalesce into a continuous film (Figure 1a). The terrace step edges act as preferential nucleation sites, which promote specific crystal orientations and suppressing twin formation (Figure 1b). Step terrace bunching leads to irregular Al island distributions across terraces. This is an effect that becomes more pronounced with increasing miscut angle and terrace number.

For the 3.5‐nm‐thick films, the morphology of the few‐atomic‐layer Al surface is strongly governed by the underlying GaAs substrate with miscut angles, resulting in an increase of RMS surface roughness with the miscut angle. Nucleation at terrace step edges also facilitates twin suppression even in such ultrathin films (as inferred in the XRD *ϕ*‐scan pattern, Figure S7b), mitigating grain‐boundary‐induced topographic features. Consequently, the RMS roughness exhibits a weaker dependence on the miscut angle over the 2°–6° range compared to those of the 0°–2° and 6°–15° ranges (Figure 6b, shaded regions of varying colors).

For the 40‐nm‐thick films, the Al surface roughness due to step terrace bunching become less pronounced, leading to a weaker yet still discernible dependence of the RMS value on the miscut angle (Figure 6a). Owing to the reduction of terrace‐induced inhomogeneities in thicker films, twin‐boundary‐suppression‐induced surface flatting becomes evident as the miscut angle increases from 2° to 6°. Notably, the 3.5‐nm‐thick film grown on the flat substrate (miscut angle = 0°) exhibits a smaller RMS value than its 40‐nm‐thick counterpart, suggesting intrinsic inhomogeneity in the Al atomic arrangement even in the absence of substrate miscut. In contrast, the 3.5‐nm‐thick film grown on the substrate with a 15° miscut angle shows a much larger RMS value due to irregular GaAs step‐terrace bunching and poorer Al crystallization.

Correlating the microstructure with room‐temperature transport properties provides a more comprehensive understanding of how film cleanliness can affect the device performance for a wide variety of applications. As discussed earlier, increasing the substrate miscut angle from 2° to 6° deteriorates crystallinity due to enhanced lattice‐mismatch‐induced strain. The resulting lattice deformation extends throughout the entire film. In thicker films (e.g., 40 nm), the atom dislocation density is relatively low, allowing twin‐boundary suppression to effectively reduce resistivity (Figure 6a). In contrast, as the film thickness is decreased to 3.5 nm, these lattice deformations become more concentrated (Figure 6b), which counteracts the resistivity reduction from twin boundary suppression and instead leads to an overall resistivity increase.

Finally, we emphasize the distinction between our substrate engineering strategy and the three commonly reported approaches for tuning superconductivity and inducing type‐II‐like behavior, which are confinement, granularity, and disorder. First, in MBE‐grown Al nanofilms, a pronounced enhancement of the critical temperature up to *T*_c_ ≈ 2.4 K has been observed as the film thickness decreases to ≈3.5 nm [22]. This enhancement may arise from surface phonon softening and the increased electronic density of states associated with geometric confinement. Moreover, when the film thickness becomes comparable to the penetration depth, magnetic flux can penetrate the film, leading to type‐II‐like superconducting behavior [60, 61]. As the channel becomes thinner, electron scattering at the surfaces and grain boundaries becomes more significant, thereby increasing the measured resistivity. Second, in granular metals such as Al, Pb and Sn, grain‐size reduction can boost *T*_c_ through density‐of‐states enhancement, again accompanied by higher resistivity relative to their single‐crystal counterparts [62, 63, 64, 65, 66]. With the loss of macroscopic coherence across grain boundaries, granular superconductors may exhibit type‐II‐like characteristics. Third, a disorder‐induced transition from type‐I to type‐II‐like superconductivity has been reported in Dirac semimetals, which is attributed to enhanced scattering and a shortened superconducting coherence length [46]. In contrast, our approach preserves a much larger film thickness (40 nm) and tunes the superconducting properties solely through the miscut angle of the substrate. The suppression of grain boundaries, reduction of normal‐state resistivity, and reconstruction of lattice strain enhance the critical current and critical field, while only slightly increasing the critical temperature. This suggests that decoherence, rather than confinement, governs superconductivity modulation in our approach. Substrate‐induced strain [67, 68, 69, 70] and engineered nanostructures such as nanomeshes [58] or nanoholes [71] have been shown to modulate superconductivity through distinct mechanisms, with potential applications including superconducting diodes [71, 72, 73, 74] and memories [38, 75]. In our work, superconductivity is tuned by precisely controlling the grain architecture via surface step‐terrace nanostructures, which reconstruct the lattice deformation during epitaxial growth. Aluminum is widely used in superconducting qubits, both in Josephson junctions and resonator circuits. For optimal qubit performance, AlO_x_ at grain boundaries, junction interfaces, and surfaces must be minimized, as it introduces microwave energy loss and superconducting decoherence [12, 13]. Although our substrate‐miscut strategy effectively suppresses Al grain boundaries, refines the crystallinity, or even enhances the superconductivity, it also introduces a new source of energy loss and decoherence through dissipative vortex motion in electric and magnetic fields. These findings indicate that the grain architecture of Al nanofilms must be carefully engineered to achieve optimal qubit performance, especially when grown on lattice‐mismatched substrates.

## 3 Conclusions

We have demonstrated that the crystalline structure of Al nanofilms can be precisely controlled using miscut GaAs substrates, where atomic step terraces act as nucleation sites for orientation‐selective growth. Increasing the miscut angle to 2° decreases the density of twin grain boundaries, smooths the surface, and enhances Al atomic ordering. Further increasing the substrate miscut angle to 6° nearly eliminates twin domains and yields micrometer‐scale single crystal with minimal surface roughness. However, the constrained domain matching imposed by narrower terraces amplifies the strain effect and deteriorates the crystallinity of the Al nanofilm. The reduction in grain boundaries mitigates charge‐defect scattering but strongly influences superconductivity. Type‐II‐like superconducting behavior, which is characterized by dissipative conduction in the superconducting state and step‐like and broadened transitions, is observed in the Al nanofilm grown on the GaAs substrate with a miscut angle of 6°. In contrast, Al on other miscut substrates exhibit well‐separated dissipation and dissipationless transport regimes, characteristic of typical type‐I superconductivity. We attribute these differences to variations in the spatial distribution of lattice deformations caused by Al/GaAs lattice mismatch stress. In the single‐crystal nanofilm, the deformation extends across the entire channel, whereas in the twinned or polycrystalline nanofilms, it is primarily confined to the domain boundaries, as confirmed by STEM and XRD analyses. The widespread distribution of atomic‐scale lattice deformations enables local penetration of magnetic flux throughout the superconducting channel, leading to type‐II‐like superconducting behavior. This study offers a novel strategy—distinct from traditional growth condition or device geometry modifications—for tailoring superconductivity, with implications for both fundamental research and practical applications.

## 4 Experimental Section

### 4.1 Material Growth

Epitaxial Al nanofilms were grown using a Varian Gen‐II III‐V solid‐source molecular beam epitaxy (MBE) system, employing a high‐purity aluminum source with a purity of 99.9998%. Semi‐insulating GaAs (001) substrates, featuring miscut angles of 0°, 2°, 6°, and 15° with respect to the [110] direction, were introduced into the MBE chamber and baked at 200°C in the entry/exit chamber for 8 h to eliminate surface moisture. Following this, the GaAs substrates were heated to 400°C for 5 h in the preparation chamber to remove any organic residues. In the growth chamber, the GaAs (001) substrates, both with and without miscut, were further heated to 600°C for 20 min to desorb the native oxide layer, and subsequently, a 200‐nm‐thick undoped GaAs buffer layer was grown at 580°C. After depositing the buffer layer, the GaAs substrates were heated again to 600°C for 3 min without arsenic flux to create a Ga‐rich surface. The substrates were then cooled down to below 0°C in the ultra‐high vacuum chamber. Following this cooling process, Al nanofilms were deposited after the residual arsenic in the growth chamber was pumped out, achieving a background pressure lower than 2 × 10^−10^ Torr. The Al nanofilms were grown at a rate of 0.1 nm/s to a thickness of 40 nm, with the chamber vacuum maintained at approximately 1–6 × 10^−10^ Torr during the growth process. The 3.5‐nm‐thick Al nanofilms were also prepared to study the Al growth dynamics on miscut substrates.

### 4.2 Material Characterization

Following the growth process, all samples were removed from the MBE chamber for subsequent characterizations. The surface morphology of the samples was analyzed using atomic force microscopy (AFM) with a Bruker Edge system operating in tapping mode. To characterize the crystal structure, a Bede D1 high‐resolution X‐ray diffractometer was employed, utilizing CuKα1 radiation (*λ* = 1.5406 Å), a two‐bounce Si 220 channel‐cut collimator crystal, and a dual‐channel Si 220 analyzer crystal. For detailed structural analysis, cross‐sectional transmission electron microscopy (TEM) and scanning TEM (STEM) specimens were prepared using a focused ion beam (FIB) method with a Hitachi NX2000 system, operated at 30 kV for initial cutting and 5 kV for final polishing. A JEOL JEM ARM 200F microscope, equipped with a Cs‐corrector and operating at 200 kV, was utilized to examine the TEM/STEM specimens. High‐angle annular dark‐field (HAADF) and annular bright‐field (ABF) STEM imaging were conducted with a convergence semi‐angle of 22 mrad and a probe size of 0.1 nm. The collection semi‐angles for the ABF and HAADF detectors were set to 10–17 mrad and 68–175 mrad, respectively, to ensure precise imaging of the sample structure.

### 4.3 Device Fabrication and Four‐Terminal Transport/Magnetotransport Measurements

MBE‐grown Al nanofilms were patterned into a Hall bar geometry using optical lithography and an etching process. The Au/Ti ohmic contacts were deposited using e‐gun evaporator and patterned using optical lithography and a lift‐off process. The Hall bar has a width of *W* = 40 µm and an arm‐to‐arm separation of *L* = 160 µm (Figure 4a). Measurements were conducted in a close‐cycle cryostat and a dilution refrigerator. A constant current was injected into the Hall bar using a Keithley 2400 (or 2612B) source measure unit while the voltage difference between the Hall‐bar arms separated by a distance of 2*L* (resulting in a channel length‐to‐width ratio of *L*/*W* = 8) was measured with a Keithley 2000 multimeter. An out‐of‐plane magnetic field was applied for magnetotransport measurements.

## Conflicts of Interest

The authors declare no conflicts of interest.

## Supporting information




**Supporting File**: smll72353‐sup‐0001‐SuppMat.pdf

## Data Availability

The data that support the findings of this study are available from the corresponding author upon reasonable request.
