# Supplementary material for: Engineering Grain Architecture in Epitaxial Aluminum on Miscut Substrates Toward Various Clean Limits and Giant Superconductivity Modulation
Source: Small. 2026 Jan 14;22(14):e12268. doi: 10.1002/smll.202512268 (PMC12965122; doi:10.1002/smll.202512268)
Supplement: Supplementary file 1 — Supporting File: smll72353‐sup‐0001‐SuppMat.pdf [file SMLL-22-e12268-s001.pdf]

Supporting Information

Engineering grain architecture in epitaxial aluminum on miscut substrates toward various clean limits and giant superconductivity modulation

Thi-Hien Do[1], Pei-Tzu Wu[2], Yu-Yao Gao[1], Ching-Hung Chen[2], Chu-Chun Wu[1], Pin-Chi Liao[3], Sung-Chieh Chiu[3], Chia-Wen Lu[3], Christos Panagopoulos[4], Atsushi Fujimori[5,6], Jenq-Shinn Wu[7], Chi-Te Liang[3,*], Sheng-Di Lin[1,*], and Shun-Tsung Lo[2,8,*]

[1]*Institute of Electronics, National Yang Ming Chiao Tung University, Hsinchu 300093, Taiwan*

[2]*Department of Electrophysics, National Yang Ming Chiao Tung University, Hsinchu 300093, Taiwan*

[3]*Department of Physics, National Taiwan University, Taipei 106319, Taiwan*

[4]*Division of Physics and Applied Physics, School of Physical and Mathematical Sciences, Nanyang Technological University, 21 Nanyang Link 637371, Singapore*

[5]*Department of Physics, University of Tokyo, Tokyo 113-0033, Japan*

[6]*Department of Physics and Center for Quantum Science and Technology, National Tsing Hua University, Hsinchu 300044, Taiwan*

[7]*Department of Electronic Engineering, National Changhua University of Education, Changhua 500208, Taiwan*

[8]*Center for Emergent Functional Matter Science, National Yang Ming Chiao Tung University, Hsinchu 300093, Taiwan*

[*]To whom correspondence should be addressed;

E-mail: ctliang@phys.ntu.edu.tw; sdlin@nycu.edu.tw; stlo@nycu.edu.tw.

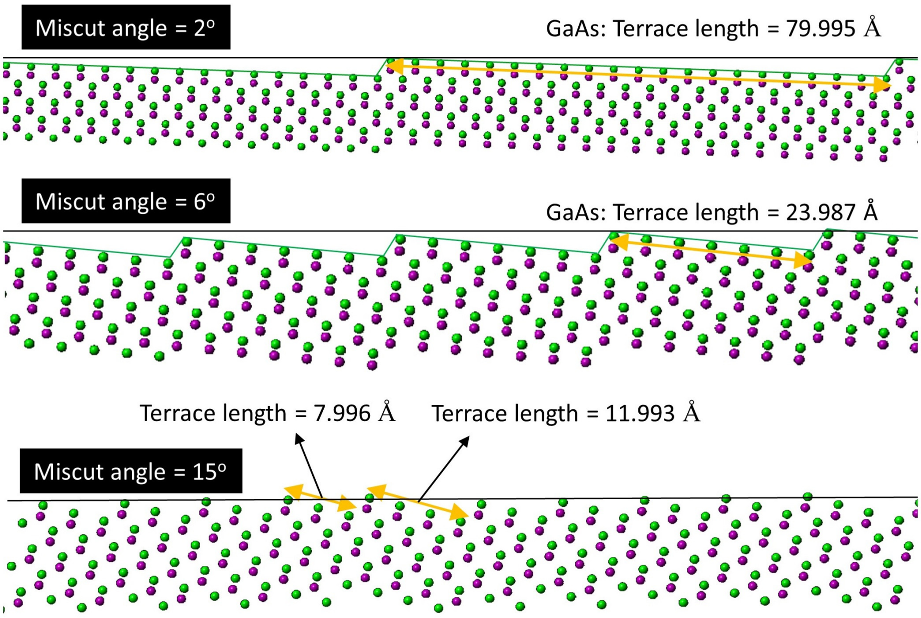

Figure S1. Surface step-terrace atomic structure of GaAs substrates with different miscut angles.

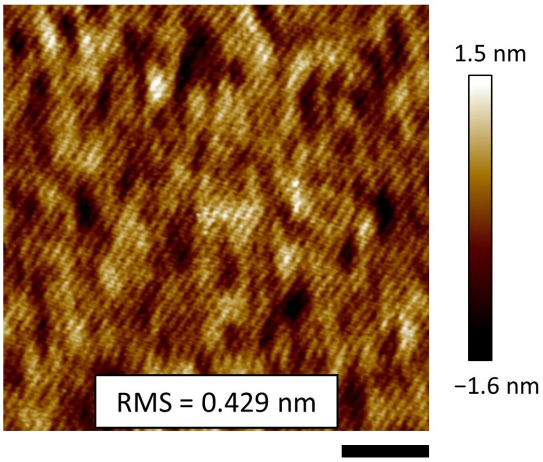

Figure S2. Atomic force microscopy image of a 6°-miscut GaAs substrate after the growth of a 200-nm-thick GaAs buffer layer (scale bar: 1 μm). The substrate surface was exposed by chemically etching away the Al thin film.

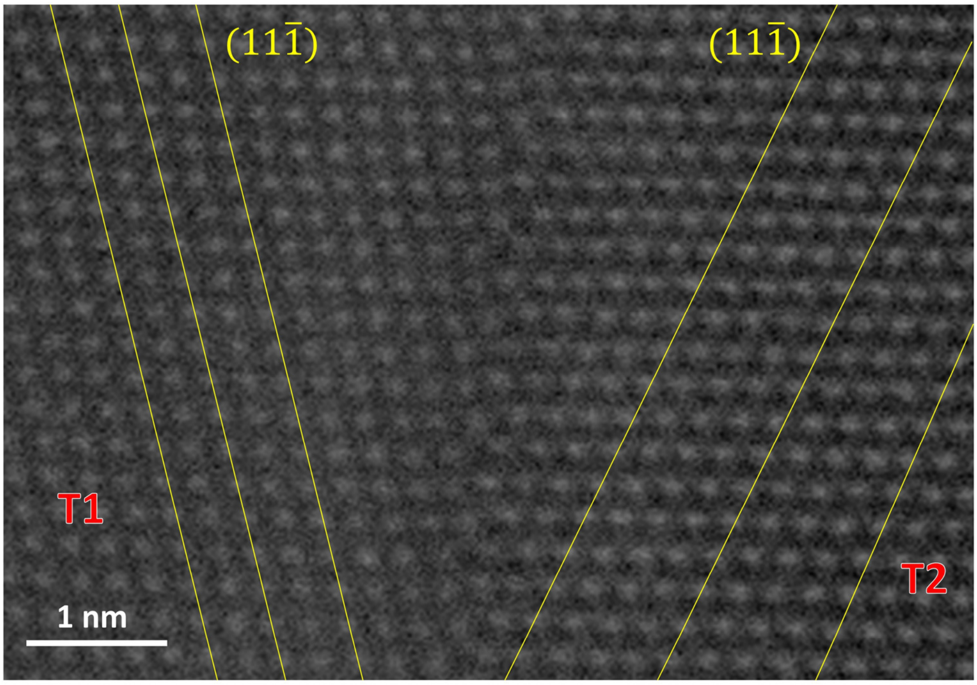

Figure S3. Zoom-in view of the high-angle annular dark-field (HAADF) scanning transmission electron microscopy (STEM) image in Fig. 2b, highlighting the twin interface between T1 and T2 crystalline domains in the Al/2°-miscut GaAs sample (denoted as sample B).

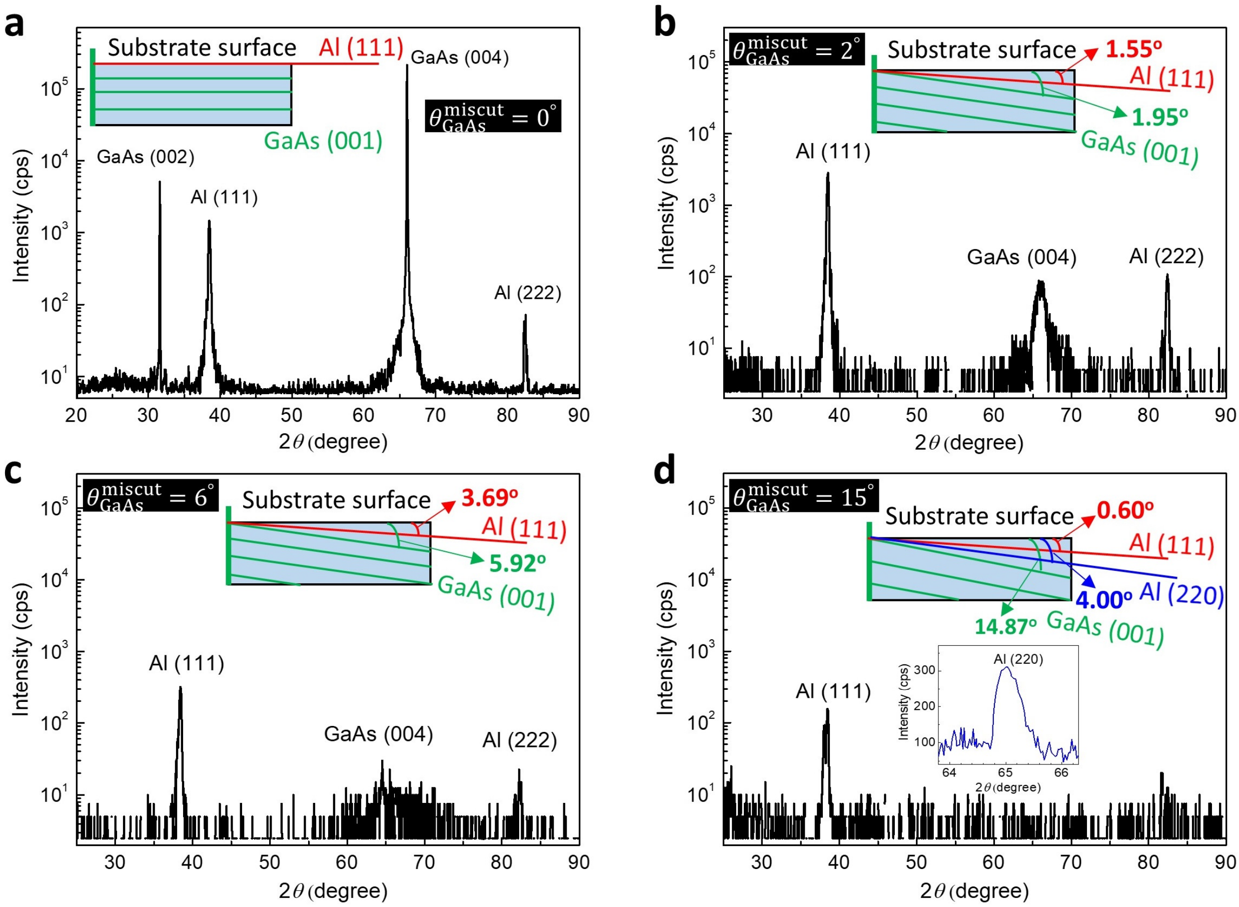

Figure S4. (**a-d**) X-ray diffraction (XRD) 2θ-θ scans for Al nanofilms grown on GaAs substrates with miscut angles of (**a**) 0°, (**b**) 2°, (**c**) 6°, and (**d**) 15°. Note that θ (2θ) corresponds to the angle between the incident X-ray beam and lattice plane (detector). Top insets, schematic diagrams indicating the off angles of Al (111) and GaAs (001) lattice planes relative to the substrate surface, as listed in Table S1. Bottom inset in (**d**), XRD 2θ-θ scan of the Al (220) reflections.

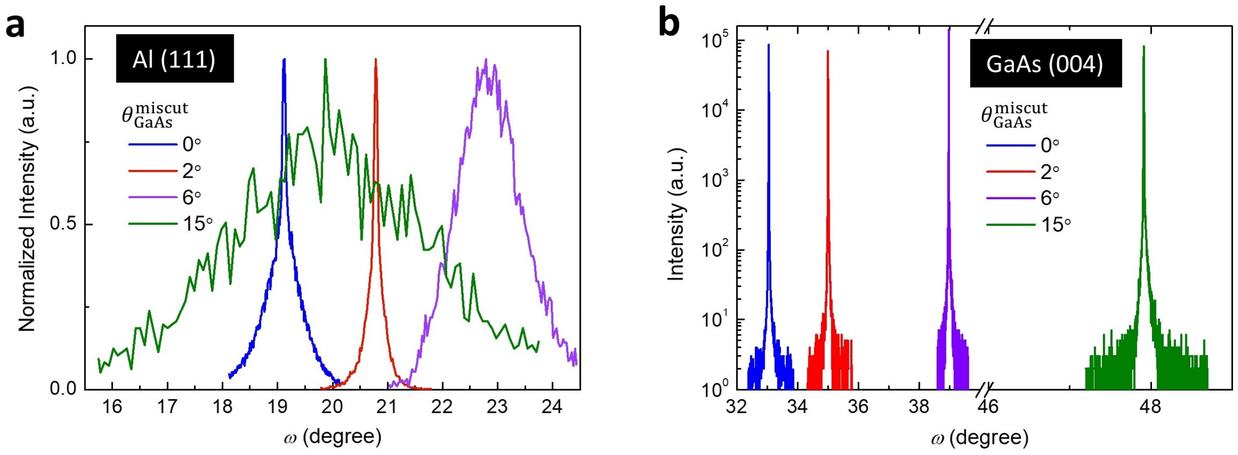

Figure S5. (**a, b**) X-ray rocking curves (from ω scans) of (**a**) Al (111) and (**b**) GaAs (001) reflections for GaAs substrates with miscut angles of θ_{GaAs}^{miscut} = 0°, 2°, 6°, and 15°. Note that ω corresponds to the angle between the incident X-ray beam and substrate surface. When $\theta_{GaAs}^{miscut} \neq 0$, θ differs from ω.

Sample (θ_{GaAs}^{miscut})	Material	ω	2θ	Off angle (θ_{GaAs}^{off} or θ_{Al}^{off})
A (0°)	GaAs (004)	33.05°	66.1°	0°
	Al (111)	19.13°	38.5°	0.12°
B (2°)	GaAs (004)	35.00°	66.1°	1.95°
	Al (111)	20.80°	38.5°	1.55°
C (6°)	GaAs (004)	38.97°	66.1°	5.92°
	Al (111)	22.94°	38.5°	3.69°
D (15°)	GaAs (004)	47.92°	66.1°	14.87°
	Al (111)<br>Al (220)	19.85°<br>36.55°	38.5°<br>65.1°	0.60°<br>4.00°

Table S1. XRD analysis of the off angles (θ_{Al}^{off} or $\theta_{GaAs}^{off} = |\omega - \theta|$) for the Al (111), Al (220), and GaAs (001) lattice planes relative to the substrate surface. The angular relationships are illustrated in the insets of Fig. S4.

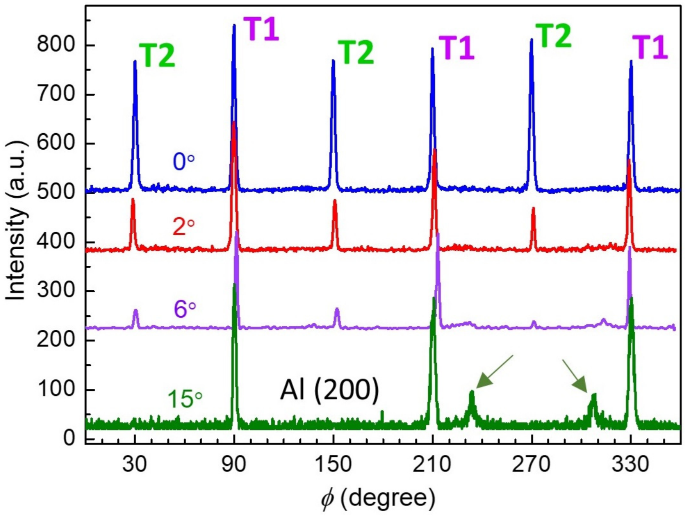

Figure S6. XRD azimuthal ϕ-scan patterns of the Al (200) reflections showing the evolution of twin crystalline domains with the substrate miscut angle from $\theta_{\mathrm{GaAs}}^{\mathrm{miscut}} = 0°$ to 15°. For the 15° substrate miscut, the Al (220) lattice plane produces additional diffraction peaks (green arrows).

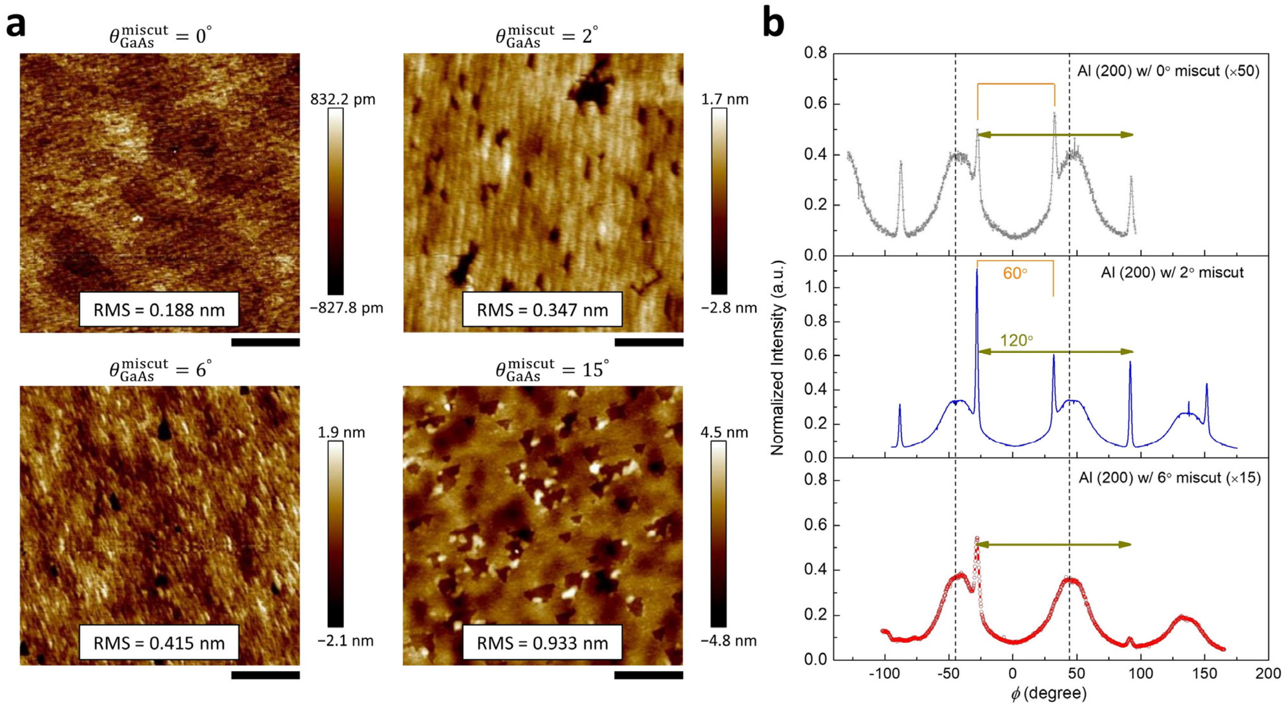

Figure S7. (**a**) Atomic force microscopy image with a scale bar of 1 μm and (**b**) X-ray diffraction azimuthal ϕ-scan pattern for the 3.5-nm-thick Al film on various miscut GaAs substrates.
